# Proteome-wide acetylation dynamics in human cells

**DOI:** 10.1038/s41598-017-09918-3

**Published:** 2017-08-31

**Authors:** Yekaterina Kori, Simone Sidoli, Zuo-Fei Yuan, Peder J. Lund, Xiaolu Zhao, Benjamin A. Garcia

**Affiliations:** 1 Epigenetics Institute, Department of Biochemistry and Biophysics, Perelman School of Medicine, University of Pennsylvania, Philadelphia, PA 19104 USA; 2 Hubei Key Laboratory of Cell Homeostasis, College of Life Sciences, Wuhan University, Wuhan, 430072 P.R. China

## Abstract

Protein acetylation plays a critical role in biological processes by regulating the functions and properties of proteins. Thus, the study of protein acetylation dynamics is critical for understanding of how this modification influences protein stability, localization, and function. Here we performed a comprehensive characterization of protein acetylation dynamics using mass spectrometry (MS) based proteomics through utilization of ^13^C-glucose or D_3_-acetate, which are metabolized into acetyl-coA, labeling acetyl groups through subsequent incorporation into proteins. Samples were collected at eight time points to monitor rates and trends of heavy acetyl incorporation. Through this platform, we characterized around 1,000 sites with significantly increasing acetylation trends, which we clustered based on their rates of acetylation. Faster rates were enriched on proteins associated with chromatin and RNA metabolism, while slower rates were more typical on proteins involved with lipid metabolism. Among others, we identified sites catalyzed at faster rates with potential critical roles in protein activation, including the histone acetyltransferase p300 acetylated in its activation loop, which could explain self-acetylation as an important feedback mechanism to regulate acetyltransferases. Overall, our studies highlight the dynamic nature of protein acetylation, and how metabolism plays a central role in this regulation.

## Introduction

**Figure 1:**
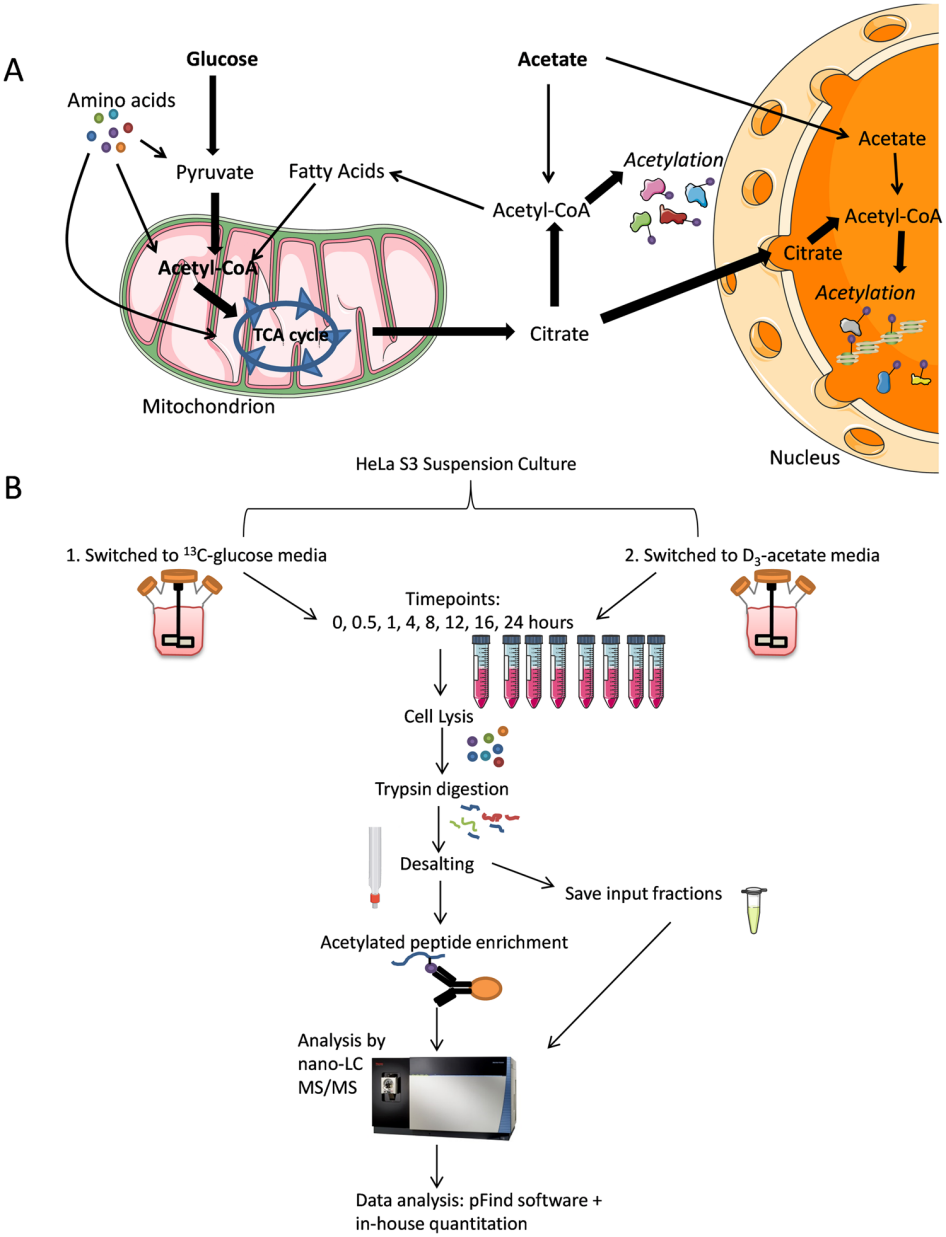
Metabolic Labeling and Workflow. (**A**) Glucose, acetate, fatty acids, and amino acids produce acetyl-CoA for use in acetylating cytoplasmic and nuclear proteins. The thicker arrows indicate that glucose contributes more to the production of acetyl-coA that subsequently acetylates proteins, compared to acetate. (**B**) The workflow consisted of growing HeLa cells in heavy-labeled media, collecting samples at eight time points, lysing the cells, digesting the proteins, enriching for acetylated peptides, and analyzing the peptides by mass spectrometry. The Orbitrap image is adapted from Thermo Fisher Scientific^56^. The cartoon cell matter and lab equipment were slightly modified from Servier Medical Art^57^.

Protein post-translational modifications (PTMs) such as lysine acetylation are critical for cell signaling, as well as for regulating protein structure and function. Lysine acetylation is the transfer of an acetyl moiety from acetyl-CoA to the ε-amino group of a specific K residue^1–3^. This acetylation is regulated by acetyltransferases and deacetylases, and thus is dynamic and reversible. Mitochondrial acetyl-CoA is produced from glucose that has been transformed into pyruvate by the pyruvate dehydrogenase complex, or by the β-oxidation of fatty acids. This mitochondrial acetyl-CoA enters into the tricarboxylic acid (TCA) cycle and produces citrate^4^, which is exported out of the mitochondria, re-converted into acetyl-CoA, and contributes to cytoplasmic acetylation as well as to acetylation of proteins within the nucleus (Fig. 1A). Acetate can also contribute to the pool of cytoplasmic acetyl-CoA, although glucose is thought to account for up to 90% of the acetyl-CoA pool under normal cell conditions^5, 6^. Other contributors to acetyl-coA production include amino acids such as glutamine, and fatty acids. However, their contribution to acetyl-coA production and subsequent acetylation is minimal compared to glucose and acetate^5–7^.

The first confident identification of protein acetylation was on histones, in the early 1960s^8, 9^. More than twenty years later, acetylation was found on a non-histone protein, tubulin^10^, and after another ten years acetylation was discovered on p53 and Tat^11, 12^. Histone acetylation is known to play a critical role in regulating chromatin accessibility and gene transcription^13–15^ in part by providing a more open chromatin structure, correlating with gene transcription, and by acting as a binding platform to recruit proteins with specialized domains to specific parts of the genome^16–18^. Recently, histone acetylation was studied using metabolic labeling of proteins with heavy ^13^C-labeled acetyl-CoA produced from ^13^C-glucose in human cells and analysis by mass spectrometry (MS)^7^. It was found that alanine production from glucose can be detected in histones if cells were grown for longer than 24 hours (i.e. new protein synthesis) on heavy glucose media. Results showed that histone acetylation has a turnover of 53 – 87 minutes. Histone acetylation is then one of the fastest PTMs in terms of dynamics; based on large-scale studies, histone acetylation has a faster turnover rate than histone methylation^19, 20^, although still slower than phosphorylation^21, 22^.

Acetylation of non-histone proteins also has many biological implications. Over 3,000 acetylation sites have been detected by large-scale proteomics studies thus far^23–25^. In addition, acetylation is an abundant modification on mitochondrial proteins, as 277 acetylation sites were identified in 133 proteins^25^. Non-histone acetylation plays a role in protein stability, DNA binding, gene expression, protein interactions, localization, mRNA stability, and enzymatic activity^26^. For example, acetylation at K709 on the transcriptional activator HIF1α by the acetyltransferase p300 leads to a decrease of polyubiquitination at this site, decreasing protein degradation and thus increasing overall protein stability^27^. Acetylation can also affect protein-protein interactions. For the transcription factor TFIIB, autoacetylation at K238 allows for increased binding to TFIIF and strong activation of transcription^28^. On the other hand, for the DNA repair protein Ku70, treatment with the deacetylase inhibitor Vorinostat leads to increased acetylation which disrupts Ku70 binding with the apoptosis regulator FLIP^29^. In some cases acetylation can affect enzymatic activity. Acetyl-CoA synthetase 2 (AceCS2) is acetylated in its active site at K642, and only upon deacetylation by SIRT3 does AceCS2 become active^30^. Importantly, aberrant acetylation can lead to a myriad of disease states including tumorigenesis, cancer cell proliferation, DNA damage, deregulation of immune response, and neurodegenerative disease^3, 26, 31, 32^. Notably, acetylation site mutations have been found by bioinformatics studies to be enriched in cancer, and integration with clinical data suggests that many of these acetylation specific mutations are associated with decreased patient survival^33^.

Due to the importance of acetylation and potential role in pathogenesis, acetylated proteins and enzymatic machinery for acetylation have been used as drug targets. Two HDAC inhibitors have received US FDA approval for treating cutaneous T-cell lymphoma patients, and many other HDAC inhibitors are currently in clinical trials^34, 35^. However, deacetylases also have many non-histone protein substrates, such as DNA-binding proteins, transcription factors, signal-transduction molecules, DNA-repair proteins, and chaperone proteins^36, 37^. A previous study used quantitative MS to identify the acetyl substrates affected by 19 different lysine deacetylase inhibitors^38^. It was found that even for proteins that shared the same deacetylase, different acetyl sites reacted differently upon inhibitor treatment in terms of the fold change increase in acetylation^38^. Thus, it is essential to not only identify acetylation sites on proteins, but to also study non-histone acetylation dynamics at the site-specific level in order to gain insight as to the role and kinetics of acetylation in biological and pathological processes.

Metabolic labeling confers several advantages for the study of site-specific acetylation dynamics. Not only does it allow for quantitative analysis for how acetyl sites incorporate acetylation, but it also provides additional information as to which metabolites contribute more efficiently to the production of acetyl-coA and subsequent installment of acetylation on a protein. Different acetyl sites even on the same protein may exhibit unique dynamics, which is detected by metabolic labeling but would not be detected by other modification tracking approaches such as stable isotope labeling of amino acids (SILAC) or pulse-chase labeling. Importantly, metabolic labeling is followed by mass spectrometry analysis, which allows for increased sensitivity in detecting acetylation at specific sites to calculate differences in acetylation incorporation between sites.

Proteomics approaches have been widely used to study the dynamics of PTMs. Histone methylation and protein phosphorylation dynamics have been studied through SILAC followed by quantitative MS^19, 21, 39^. Histone acetylation dynamics have been studied by radiolabeling^40^, and by metabolic labeling followed by quantitative MS^7^. In addition, the use of antibodies specific for acetylated lysine has proved vital for enrichment of acetylated peptides and improved subsequent detection of acetylation sites^41^. The importance of understanding the dynamics of non-histone proteins is illustrated by the numerous critical biological functions that are implicated by acetylation, and yet protein acetylation dynamics have previously not been well-characterized. In this study, a quantitative proteomics approach was applied to determine acetylation dynamics of non-histone proteins, which allowed us to confidently estimate the turnover rate of more than 1,000 acetylated peptides generated via acetyl-CoA produced from different metabolic pathways.

## Results

**Figure 2:**
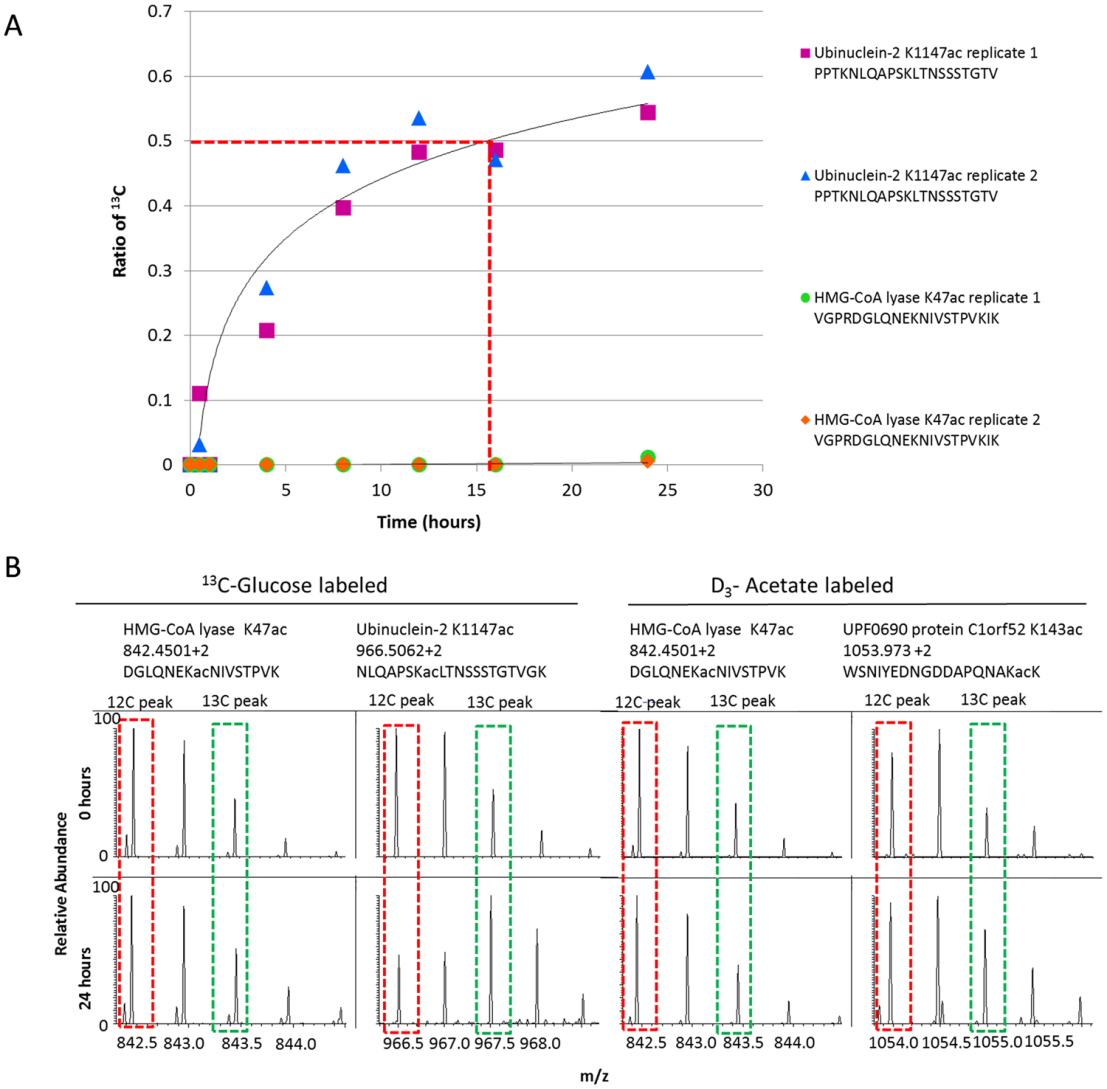
Half-Life of Acetylation Incorporation. (**A**) The ratio of heavy acetylation incorporation ^13^C/(^13^C + ^12^C) plotted against the time. The half-time of heavy acetyl incorporation was calculated by fitting the ratios of ^13^C/(^13^C + ^12^C) acetylated peptides at each time point to the equation y = a*(1-e^(-x/b)). In the case if after 24 hours the amount of heavy labeled acetylation did not reach 0.5, as shown for the HMG-CoA lyase peptide, we used the linear polynomial equation y = ax + b. The red dashed line indicates the half-life for the Ubinuclein-2 K1147 acetyl site. (**B**) The isotopic patterns of heavy acetylation incorporation for two peptides identified in the ^13^C-glucose labeling experiment, and two peptides in the D_3_-acetate labeling experiment are shown. The top row shows the mass spectra for the 0 hour time point, and the bottom row shows the mass spectra for the 24 hour time point. For each glucose and acetate labeled sections, the left spectra indicate a peptide that did not incorporate heavy acetylation, and the right spectra indicate a peptide that did incorporate heavy acetylation over time. The red dashed box indicates the monoisotopic peak, and the green dashed box indicates the heavy labeled peak.

Both ^13^C-glucose and D_3_-acetate are metabolically converted into heavy acetyl-CoA and subsequently incorporated into proteins, albeit through different metabolic pathways (Fig. 1A). To track acetylation dynamics, HeLa cells (in duplicate cultures) were incubated with either ^13^C-glucose or D_3_-acetate, which serve as carbon sources for protein acetylation (Fig. 1B). Glucose and acetate were chosen as metabolic sources for this study since they are the major contributors of acetyl-coA production^5–7^, and this study aimed to gain a comprehensive analysis of acetylation dynamics on the proteome-scale. Samples were collected at various time points. The cells were lysed and proteins trypsin digested. Acetylated peptides were then enriched using anti-acetyl-lysine antibodies^41^ and run on nano liquid chromatography coupled online with tandem mass spectrometry (nanoLC-MS/MS) on an Orbitrap Fusion mass spectrometer (Thermo Fisher Scientific), which allows for detection of isotope incorporation into acetylated proteins. Quantification of antibody enriched acetylated peptides was performed using an in-house developed tool to extract isotopic patterns. Importantly, for the numbering of residues to calculate the acetyl site position, the initiator methionine at the start of the sequence was not counted, and thus the acetyl positions may be one residue less than reported in other studies or UniProt. The half-time of heavy acetyl incorporation was calculated by fitting the ratios of ^13^C/(^13^C + ^12^C) acetylated peptides at each time point to the equation y = a*(1-e^(-x/b)) (Fig. 2A). For the determination of the half-life, y was set as 0.5, and x was obtained after fitting the curve based on data obtained from all time points. In the case if after 24 hours the amount of ^13^C labeled acetylation did not reach 0.5, the linear polynomial equation y = ax + b was used to estimate half-time. Peptides that did not incorporate any heavy labeling by 24 hours were also observed (Fig. 2B and Supplementary Figure S3).

### Quality Control

The ratios of ^13^C/(^13^C + ^12^C) acetylated peptides showed an overall steady increase across the time points as displayed by the principal component analysis (PCA, Supplementary Figure S1). Data from the heavy glucose labeled cells showed a better trend than data from the heavy acetate labeled cells, indicating that heavy acetyl incorporation is less marked in this acetate experiment. This was also expected, since acetate is a lesser-used metabolite by the cell for the production of acetyl-CoA^5, 6^. We also compared the results from the different time points using multi-scatter plotting and Pearson correlation; such results confirmed higher correlation between closer time points (Supplementary Figure S2).

**Figure 3:**
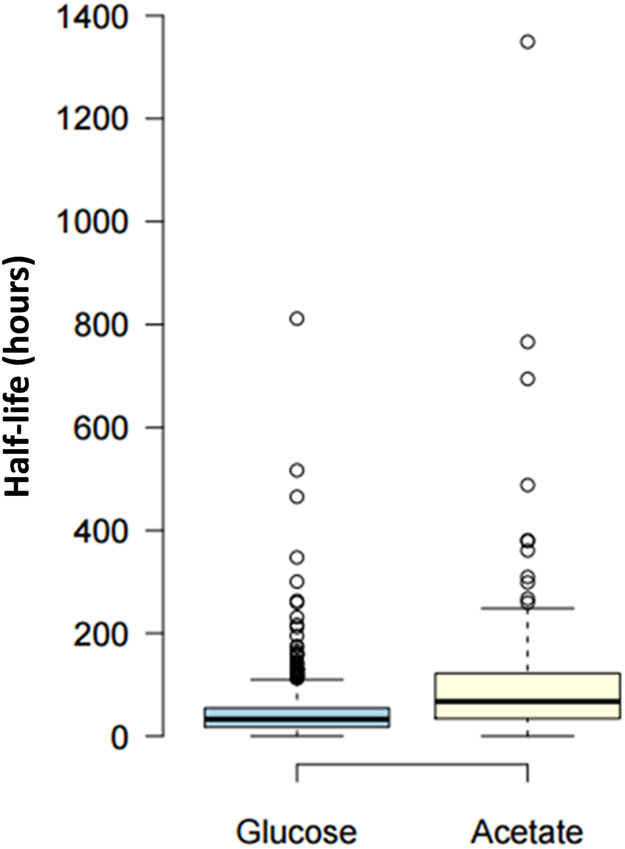
Boxplot of Acetylation Half-Lives. The boxplot portrays the half-lives of acetylation for peptides labeled by glucose, and half-lives of acetylation for peptides labeled by acetate. Only peptides with a statistically significant increasing acetylation incorporation trend are shown.

Overall, 4,994 acetylated peptides were detected, of which 2,375 acetylated peptides were identified from the cells labeled with ^13^C-glucose and 2,619 acetylated peptides from the cells labeled with D_3_-acetate (Table 1). For the glucose labeling experiment, 1,814 acetylated peptides were significantly correlated between biological replicates. For the acetate labeling experiment, 2,100 acetylated peptides were significantly correlated between replicates. Results showed that out of 1,821 acetylated peptides detected by glucose labeling, 901 peptides had a significantly increasing acetylation trend. Significance of acetylation incorporation as monotonic trend across the time points was determined by the Mann-Kendall statistical test (p-value < 0.05). On the other hand, only 126 out of 2,120 peptides from the acetate labeling experiment had a significantly increasing acetylation trend. This suggests that in this cell model, glucose is the major contributor to acetyl-coA, which is consistent with previous work that has reported that glucose generates as much as 90% of acetyl-CoA under cell culture conditions^5, 6^. Considering the results at the protein level, we identified for the ^13^C-glucose labeling experiment 702 unique proteins with significant acetylation increase across time points. For the D_3_-acetate labeling experiment, only 70 unique proteins with significant acetylation trends were detected (Table 1). In this study we define acetylation half-life as the amount of time for the ^13^C/(^13^C + ^12^C) ratio to reach 0.5. The heavy acetylation incorporation half-life showed a large range for the peptides detected (Fig. 3). The heavy acetylation incorporation half-life for peptides labeled by glucose ranged from 0.2 hours to 811 hours (estimated), while the half-life heavy acetylation incorporation time for peptides labeled by acetate ranged from 0.04 hours to 1349 hours (estimated). Overall, observations confirmed that acetylation by glucose labeling has a much faster half-life incorporation rate than by acetate labeling, and that our method allows for the determination of the turnover rate of thousands of acetylated sites on a global proteome scale.

**Table 1:** Counts of Acetylation Identifications.

Important Numbers	Overall	Glucose	Acetate	Labeled in Both
Acetylated peptides detected	4994	2375	2619	1820
Heavy-labeled acetylated peptides	3941	1821	2120	1378
Acetylated peptides with a statistically significant trend	1027	901	126	48
Acetylated proteins	2206	1044	1162	795
Acetylated proteins with a statistically significant trend	772	702	70	48
Peptides (statistically significant trend) correlated between replicates	490	485	5	2
Correlated between glucose and acetate	14			

The counts of the identified acetylated peptides and proteins in the glucose and acetate labeling experiments are displayed. A “statistically significant trend” means that for these acetylated peptides, the p-value for the Mann Kendall statistical test was less than 0.05. Peptides that are counted as “correlated between replicates” means that the p-value of correlation for these peptides was less than 0.05. “Labeled in both” means that those acetylated peptides were detected in both the ^13^C-glucose labeling experiment and the D_3_-acetate labeling experiment.

### Acetylation Half-life Rates Form Distinct Clusters

**Figure 4:**
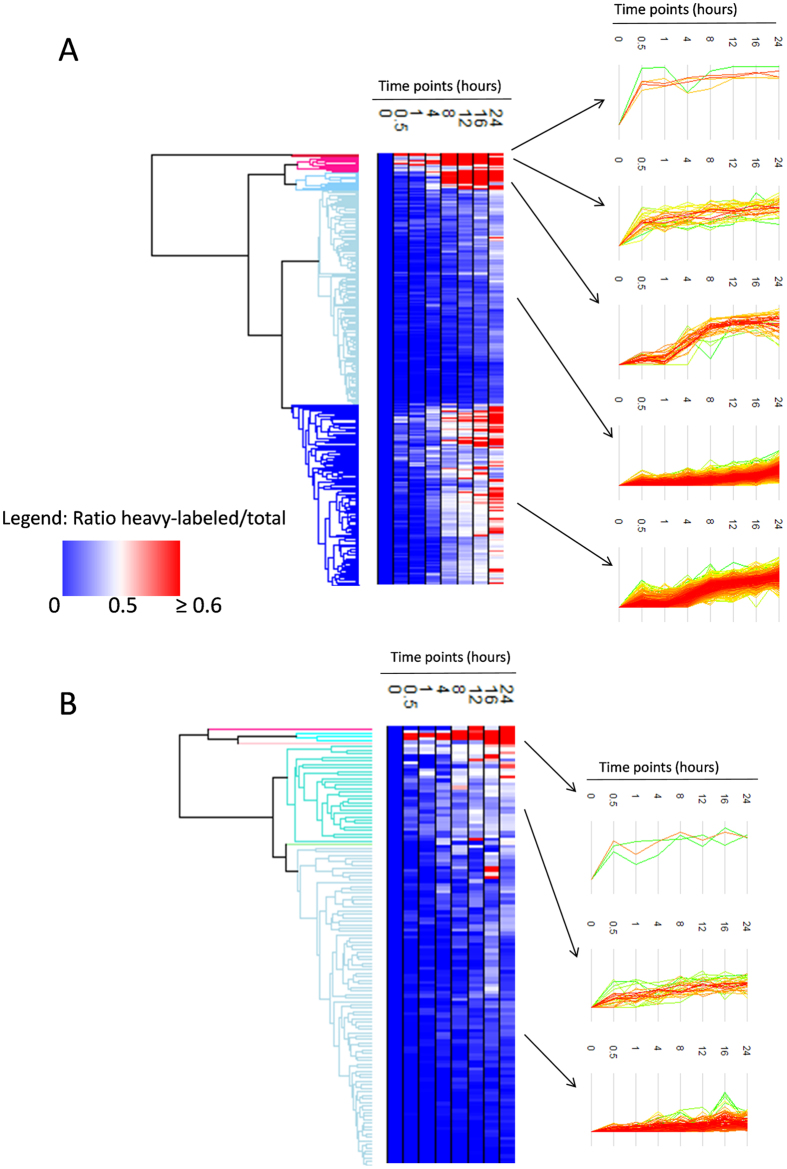
Clustering of Heavy Acetylation Incorporation Trends. (**A**) The heatmap demonstrates the ^13^C/(^13^C + ^12^C) ratio at each time point for the peptides that showed statistically significant heavy acetylation incorporation in the ^13^C-glucose labeled experiment. The ratios are an average between two replicates. The trends of heavy acetylation incorporation are indicated by the graphs on the right. (**B**) The heatmap demonstrates the ^13^C/(^13^C + ^12^C) ratio at each time point for the peptides that showed statistically significant heavy acetylation incorporation in the D_3_-acetate labeled experiment.

To group acetylation events into fast and slow rates, we organized our data using hierarchical clustering and displayed them as heatmaps using Perseus^42^. Heatmaps were created using only peptides with significant incorporation of heavy acetylation (Mann Kendall test, p-value < 0.05). The heatmap displayed five distinct clusters of acetylation incorporation for the peptides that had been labeled by ^13^C-glucose, identifying acetylated peptides that have a fast half-life for acetylation incorporation, medium half-life, and comparatively slower half-life (Fig. 4A). The clusters were applied to the GOrilla software^43^ to calculate functional enrichment of proteins belonging to each subgroup as compared to all the proteins present in the heatmap. Acetylated peptides with the second fastest heavy acetylation incorporation half-life were slightly enriched in proteins that function in chromatin organization (Supplementary Figure S7A), while acetylated peptides with medium heavy acetylation incorporation half-life were slightly enriched in RNA metabolic processes (Supplementary Figure S7C). Acetylated peptides with the slowest acetylation incorporation half-life were slightly enriched in proteins that function in cellular lipid metabolic processes (Supplementary Figure S7B). For the D_3_-acetate labeling, 3 major clusters are observed, identifying acetylated peptides that have fast, medium, and slow half-life of heavy acetylation incorporation (Fig. 4B). However, for these clusters identified for the D_3_-acetate labeling, functional enrichment was not observed for any of the clusters by using GOrilla software. Together, the data shows that acetylation turnover rates are not drastically classified into proteins with specific functions or that belong to specific compartments. However, chromatin related proteins and RNA metabolic proteins were found to be favored in terms of rapid acetylation turnover as compared to other functions.

Next, we transformed the trends obtained from the ^13^C/(^13^C + ^12^C) ratio for all time points into a cumulative trend. Specifically, the cumulative values were calculated by normalizing each ratio considering the maximum value of the row as 100%, so that the amount of acetylation at the 24 hour time point is 1, and thus these cumulative heatmaps display at which time point the acetylation site begins its turnover. Peptides with equal turnover rate at every data point would generate a straight line, while peptides with delayed incorporation would display a hyperbolic curve. Four distinct clusters of acetylation incorporation are observed for the cumulative values of the peptides for both the glucose labeling and the acetate labeling, indicating four different trends of acetylation incorporation in each of these conditions (Supplementary Figure S4). The two heatmaps were much more alike than the two heatmaps obtained using the raw ratio values (Fig. 4). This indicated that, despite the acetate labeling leading to a much slower incorporation of heavy acetylation, both glucose and acetate are processed by the cell during the same time frame. If, for instance, acetate would be used by the cell only in absence of glucose, we would have observed only hyperbolic trends in the heatmap displaying cumulative rates of acetate labeling samples.

We then combined the average and cumulative results for the peptides found to have significant incorporation of acetylation in both glucose and acetate labeled samples (Supplementary Figure S5). These heatmaps included 48 peptides, which showed similar rates of acetylation incorporation by both glucose labeling and acetate labeling. This is unexpected because the overall trend for the total peptides detected is that heavy acetylation incorporation is slower by acetate labeling. Together, this analysis revealed that overall significant differences can be observed between peptides in terms of acetylation turnover, and that only a small subsection of peptides have shared trends when glucose and acetate are adopted by the cell to generate acetyl-CoA.

### *In Silico* Simulation of Acetylated Protein Networks

**Figure 5:**
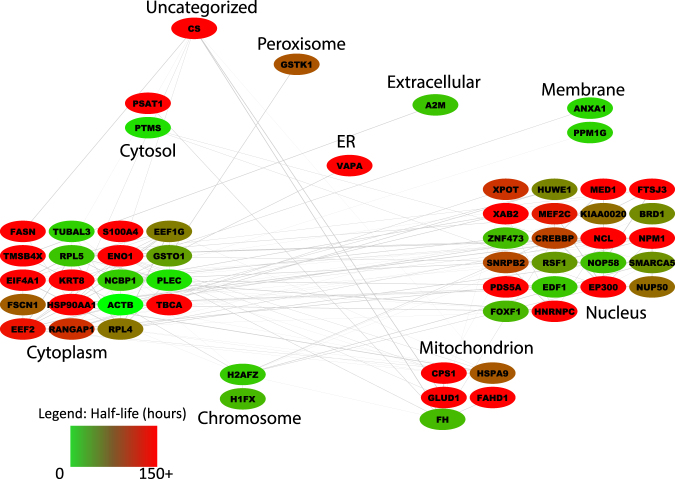
Acetate-Labeled Networks. The map portrays the connections between proteins with acetyl sites identified in the D_3_-acetate labeled experiment. The connector lines are based on known protein-protein interactions retrieved from STRING^53^. The color indicates the average half-life between all detected sites within the protein.

Since we could not identify strong functional enrichment of proteins with fast or slow acetylation rates, we grouped all proteins with significant acetylation turnover (Mann Kendall test, p-value < 0.05) and analyzed them as protein-protein interaction networks. To simplify the visualization we divided the proteins into cell compartments, in agreement to their UniProt-reported localization (Fig. 5 and Supplementary Figure S6). Proteins found to not have any known interaction with other proteins in the dataset were not displayed, facilitating the focus on proteins that have known annotations. Proteins were color coded by the average acetylation half-life of all the acetylation sites detected in the protein. For proteins labeled by ^13^C-glucose, the majority of acetylation had half-lives of less than 150 hours, while for the D_3_- acetate labeling the majority of the acetylated proteins had acetylation half-lives of greater than 150 hours. Most of acetylated proteins detected in both the glucose and acetate labeling experiments localize to the cytoplasm or nucleus. By glucose labeling, there appears to be a slight enrichment of proteins with fast acetylation half-lives at the membrane, and a slight enrichment of slow acetylation half-lives in the mitochondria. For acetate labeling, the sample size is too small to see any particular enrichment.

### Quantification of Heavy Acetyl-CoA from Heavy Glucose and Heavy Acetate Cultures

**Figure 6:**
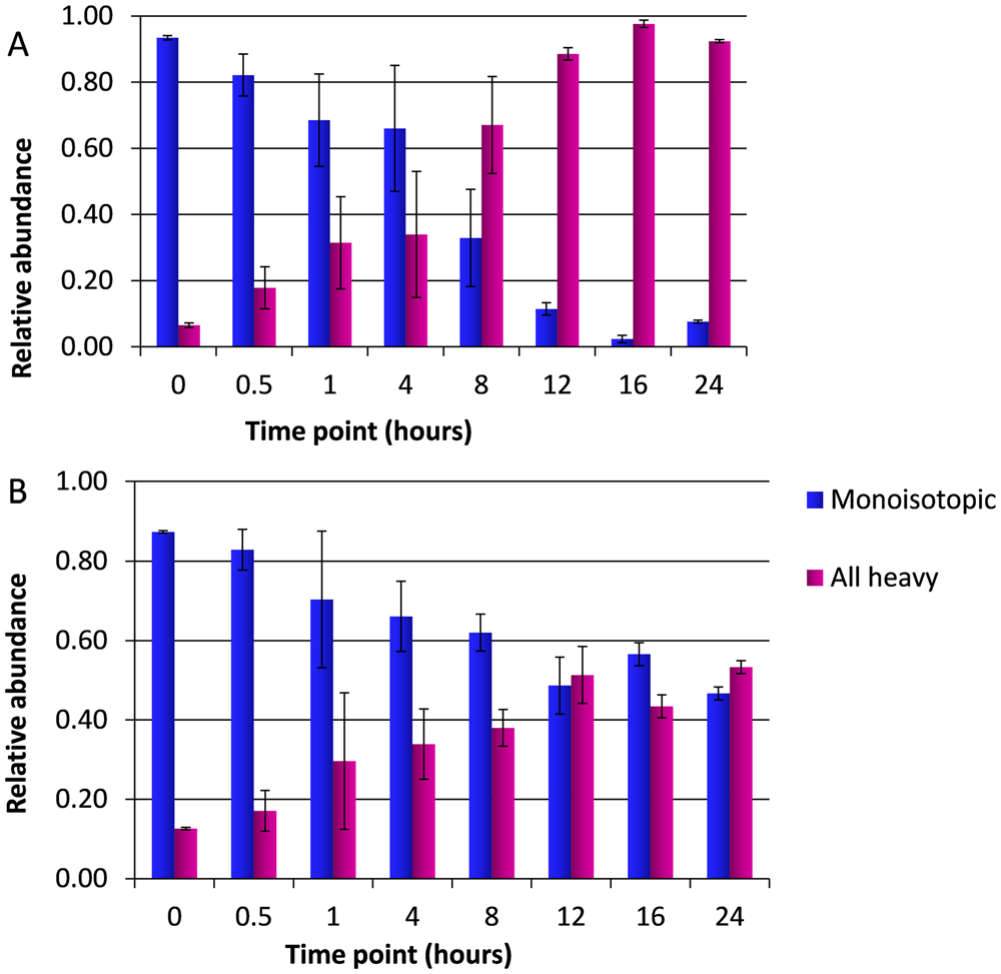
Acetyl-CoA Metabolic Labeling. (**A**) Acetyl-CoA extracted from HeLa cells grown in ^13^C-glucose. The relative abundance of the monoisotopic and heavy labeled peaks of acetyl-coA at each time point is plotted. The relative abundance is calculated from two replicates, with the standard deviation indicated. (**B**) Acetyl-CoA extracted from HeLa cells grown in D_3_-acetate. The relative abundance of the monoisotopic and heavy labeled peaks of acetyl-coA at each time point is plotted. The relative abundance is calculated from two replicates, with the standard deviation indicated.

In order to determine whether the differences in acetylation rates is due to the slower conversion of acetate into acetyl-CoA or rather in the unavailability of acetyl-CoA generated from the metabolism of acetate, we quantified the rate of production of heavy acetyl-CoA in our cell models using LC-MS based metabolomics. HeLa suspension cells were prepared in the same manner as previously described; ^13^C-glucose or D_3_-acetate were introduced into HeLa cell cultures, in duplicate, and samples were collected at eight time points. Metabolites were extracted with 80% methanol/20% H_2_O and analyzed by LC-MS on a LTQ Orbitrap mass spectrometer (Thermo). From two replicates labeled by ^13^C-glucose, it was observed that the relative abundance of the monoisotopic peak for acetyl-CoA decreases with increasing time points, whereas the relative abundance of the total heavy labeled peaks increases over time (Fig. 6A). The switch to more heavy labeled acetyl-coA occurs at about 8 hours. For the cells labeled by D_3_-acetate, there is a slight decrease in the relative abundance of the monoisotopic peak of acetyl-coA over time, and a slight increase in the relative abundance of heavy labeled acetyl-coA peaks over time (Fig. 6B). However, in the ^13^C-glucose labeled cultures, the relative abundance of the heavy labeled acetyl-coA peaks reach almost 100% by 24 hours, while the D_3_-acetate labeled cultures only reach about 50% relative abundance by 24 hours (Fig. 6). There does not appear to be a definite switch from majority of monoisotopic peak to majority heavy labeled acetyl-coA until the 24 hour time point for the D_3_-acetate labeled cells. The ^13^C-glucose labeled cultures therefore generate heavy labeled acetyl-coA at about four times the rate that D_3_-acetate labeled cultures generate acetyl-coA, which may be a primary determinant of why heavy acetylation incorporation via D_3_-acetate labeling is slower.

### Protein Acetylation Sites Map to Specific Domains

**Figure 7:**
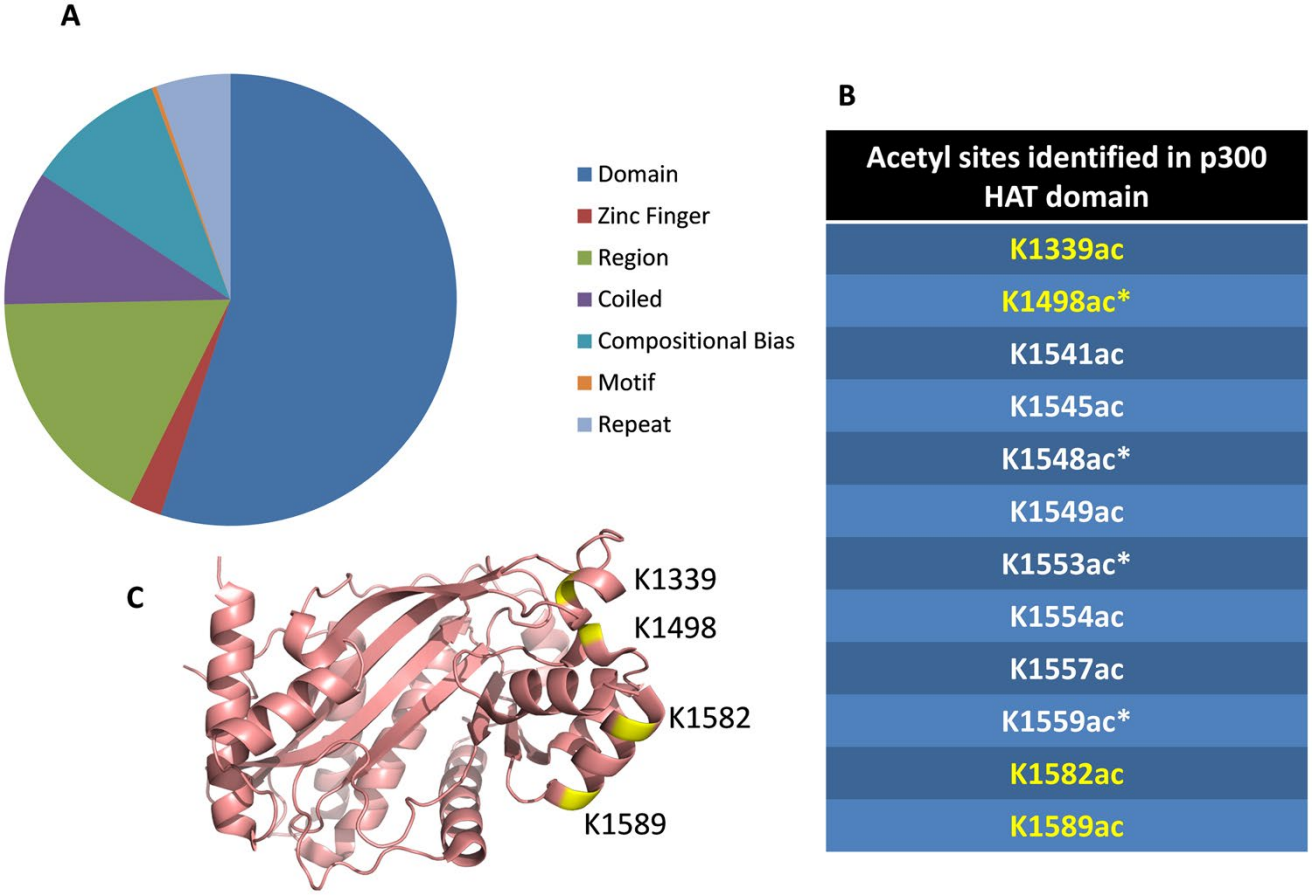
Domain Analysis for Acetylated Peptides. (**A**) A list of annotated domains from UniProt was used to map the acetyl sites identified in this dataset to regions with specific functions, and a total of 300 acetyl sites mapped to specific domains. The full list is in the supplementary material (Supplementary Table S2). (**B**) Twelve acetyl sites detected in this study for p300 fall within the histone acetyltransferase (HAT) domain. Those highlighted in yellow map on the structure, and the starred sites have previously been reported to occur due to autocatalysis. (**C**) The previously solved structure of the HAT domain of p300^58^ with four of the identified acetyl sites from this study highlighted in yellow.

With this dataset, a program was developed to automatically map whether detected acetyls were within known protein domains. Motif analysis was conducted on the acetylation turnover clusters observed, however no sequence motif was found to be significantly enriched for any turnover group. On the other hand, a depletion of histidine residues was typical of all acetylated peptides detected by glucose labeling (Supplementary Figure S8A). We then retrieved the annotated domains of all identified proteins from the UniProt database. From here, we mapped the sites identified in our dataset into regions known to have a specific function in the respective proteins. We identified in total 300 acetyl sites that mapped to specific domains (Fig. 7A), including zinc finger domains, coiled domains, compositional biases (areas enriched in certain amino acids), motifs, and repeats. One of the identified domains was the histone acetyltransferase domain (HAT) of p300, where multiple acetyl sites were mapped (Fig. 7B and C). These acetyl sites that mapped to domains provide additional information on how acetylation may influence function in these proteins.

## Discussion

We have presented the most comprehensive study of protein acetylation dynamics to date. In terms of half-lives for heavy-labeled modification incorporation, non-histone acetylation has the largest half-lives (around 69.5 hours for sites labeled by glucose), followed by histone methylation (around 5 hours)^19, 20^, followed by histone acetylation (53-87 min)^7^, followed by non-histone phosphorylation (about 20 minutes)^21, 22^.

An explanation for the acetyl sites that are exceedingly slow may be that they are a result of non-enzymatic chemical acetylation. A previous study has shown by Nucleic Acid Programmable Protein Array and validation by MS that *in-vitro* with physiological acetyl-CoA concentrations, 58 out of 6,000 proteins were identified as non-enzymatically acetylated^44^. This study suggests that non-enzymatic acetylation is not a fast process, since they used a larger than physiological concentration of acetyl-coA (300 uM) for 90 minutes to overnight incubations in order to successfully detect non-enzymatic acetylation. The data was compared to published proteomics data, and 28 out of the 58 proteins were also observed to be acetylated *in-vivo*. This study observed non-enzymatic acetylation of ribosomal subunit RPS26 at K65, an acetyl site that was detected in our study as well, further supporting the idea of potential non-enzymatic acetylation occurring. In our dynamics study the time to incorporate 50% heavy acetylation was found to be about 24 hours for RPS26 K65ac by glucose labeling. Although this is not the slowest heavy acetylation incorporation time that was observed, it does support the idea that some of the slow incorporations of heavy acetyl could be a result of non-enzymatic acetylation.

Another study calculated the chemical reactivity of 90 sites in 8 purified proteins by incubation with acetyl-coA, followed by analysis by MS which allowed for site-specific acetylation stoichiometry quantification^45^. This study observed that even after 1 hour incubation with concentrations of acetyl-coA that varied up to 8 mM, some sites displayed very low acetylation stoichiometry and reactivity. For mitochondrial glutamate dehydrogenase (GDH), the rate constants for non-enzymatic acetylation of fifteen acetyl sites were calculated, and K502 displayed the second highest reactivity when incubated with acetyl-coA. Our study detected K502 acetylation of GDH, in the both glucose and acetate heavy labeling experiments, and the heavy acetylation trend for this site was statistically significant by acetate labeling. The high reactivity of K502 for non-enzymatic acetylation may explain why this was the only statistically significant site identified in our study: K502 non-enzymatic acetylation is a more likely event than non-enzymatic acetylation of other sites that display lower reactivity. K502 of GDH displayed a large heavy acetylation incorporation half-life of 80.3 hours in the acetate labeling experiment. It is important to consider that the current data is calculated from *in-vivo* acetylation happening in the cell, and as such, there are other factors to take into consideration that may result in non-enzymatic acetylation being slower than observed *in-vitro* (i.e. protein conformation, localization, etc). The time necessary to metabolically produce enough acetyl-coA for non-enzymatic acetylation to occur may also affect the rate of non-enzymatic acetylation *in-vivo* as well. Thus, the acetyl sites that are exceedingly slow, especially in the mitochondria, may be due to non-enzymatic acetylation.

The dynamics results presented in the current study provide interesting correlations when compared with the results of a previous large scale study that evaluated the specific acetyl sites affected by 19 different lysine deacetylase inhibitors (KDACI). The most selective inhibitor in the screen was found to be PCI34051, an HDAC8 inhibitor^38^. SMC3 was found to be the key target of this inhibitor, with acetylation of K105 and K106 increasing upon inhibition of HDAC8. These acetyl sites were most affected by HDAC8 inhibition, which correlates well with our dynamics data. These sites were detected in the glucose labeling experiment and displayed fast heavy acetylation half-lives comparatively to other acetyl sites. Sites with faster acetylation incorporation are expected to show a more dramatic effect upon deacetylase inhibition. As mentioned earlier, the acetyl positions in our study may be one residue less than reported in other papers. SMC3 K104 (reported as 105 in other studies) was found as a unique acetylation, and in combination with K105ac (106ac). The half-life for heavy acetylation incorporation for K104 was calculated to be 15.94 hours. The KDACI study also evaluated nicotinamide, a noncompetitive inhibitor of sirtuins. Proteins with acetyl sites upregulated upon nicotinamide treatment included known substrates of sirtuin 1, such as nucleophosmin (NPM1), x-ray repair cross-complementing protein 6 (Ku70), and X-ray repair cross-complementing protein 5 (Ku80). Our study detected 5 acetyl sites on NPM1, four of which have a relatively fast turnover, with half-lives under 30 hours. Dynamics for acetyl sites on Ku70 and Ku80 were also calculated in the current study by glucose labeling, with K410 of Ku70 demonstrating a heavy acetylation incorporation half-life of 40.85 hours, and K564 on Ku80 displaying a faster half-life of 16.58 hours. Ku80 displays more dynamic acetyl sites, and was also more affected by nicotinamide treatment, with a larger fold change in acetylation upon inhibitor treatment. These examples support the conclusion that more dynamic acetyl sites are more affected by inhibitor treatment.

In addition to identification of acetylation sites and analysis of dynamics, this current study also mapped identified acetylation sites to known protein domains, coiled regions, motifs, and regions of compositional bias. For example, twelve unique sites were identified within the histone acetyltransferase (HAT) domain of p300, with many of these sites found in combination. The HAT domain of p300 is responsible for acetylating a wide variety of substrates through a ping-pong mechanism^46^. Four out of the twelve acetyl sites on p300 identified in our study have been previously identified to be caused by autocatalysis of p300, and necessary for p300 activation to acetylate other substrates^47^. Eight of the detected sites on p300 fall within the loop region, residues 1520-1560, which has been demonstrated to be a flexible and proteolytically sensitive region^47^. Dynamics data of the acetylation sites within the HAT domain of p300 is critical, as acetylation within this domain has been demonstrated to be an essential regulator of p300 activity. A hypoacetylated construct of p300, with fewer than two acetylation events, was calculated to have a V_max_/K_m_ that is four-fold lower than the fully acetylated recombinant protein. Autoacetylation of the construct stimulated the domain and increased activity 11-fold^47^. This suggests that acetylation of the flexible loop acts as a regulatory mechanism: the loop blocks active site of the HAT domain in p300, is autoacetylated and released so that the active site is not blocked anymore, and p300 is activated to acetylate other substrates. The dynamics results showed that the heavy acetylation incorporation rates for these sites were all fast compared to sites on other proteins identified. Specifically for glucose labeling, some acetyl sites of p300 displayed a half-life of heavy acetylation incorporation at as low as 5 hours. This suggests that the regulatory mechanism of autoacetylation of the autoinhibitory flexible loop region is quick. Given that p300 is known to acetylate many different substrates that affect a variety of biological processes, it makes sense that its regulation mechanism would be more quick and transient than an acetyltransferase with only a couple of targets. As exemplified by the acetyl sites mapped to the HAT domain of p300, mapping the acetyl sites identified adds an extra layer of insight to the data on the dynamics of these sites.

Since it was found that overall ^13^C-glucose labeled cells exhibit faster heavy acetylation incorporation rates than D_3_-acetate labeled cells, metabolite analysis was conducted to determine whether a similar trend was observed for acetyl-coA heavy labeling. Metabolite analysis showed that heavy labeling of acetyl-coA by ^13^C-glucose occurs at a faster rate compared to heavy labeling by D_3_-acetate. Acetate is converted to acetyl-coA by acetyl-coA synthetase^4^. On the other hand, generation of acetyl-coA from glucose is not a one-step process^5^. Even though glucose goes through more metabolic steps to produce acetyl-coA, it still generates acetyl-coA faster than acetate generates acetyl-coA. One possible explanation is that since glucose is thought to account for up to 90% of the acetyl-CoA pool under normal cell conditions^5, 6^, just the large difference in the amount of glucose versus acetate being used to produce acetyl-coA is contributing to the difference in rates of production. A study in rat hepatocytes found that in the cytoplasm, the rate of acetyl-coA synthesis was lower than that of acetyl-coA hydrolysis^48^, indicating that the net flux in the process was in the direction of the reactant, acetate. This suggests that the production of acetyl-coA from acetate is not as efficient as the production of acetyl-coA from glucose. The differences in the rates of acetyl-coA production by acetate and glucose as indicated by the metabolite analysis aligns well with the patterns in acetylation dynamics observed in this study. Since acetate contributes less to the acetyl-coA pool and at a considerably slower rate than glucose contributes to acetyl-coA, it is expected that proteins acetylated from acetate-labeled acetyl-coA would exhibit much slower incorporation of the heavy acetyl group, and that there would be less proteins with labeled acetylation detected from acetate-labeled cells. Thus, due to these metabolic processes and the cell’s preference for glucose, glucose labeling is more efficient for gaining insight on the acetylation dynamics of a large range of proteins. In the future, it would be also informative to get differential acetyl-coA quantification from different organelles, but currently technical challenges are still excessive^49^.

In summary, our results from metabolic labeling followed by MS provide insight as to the dynamics of specific acetylation sites and how efficiently metabolites such as glucose or acetate are incorporated into acetylation of proteins. Mapping the acetyl sites to specific domains of proteins allows for further understanding of how these acetyl marks affect the function of the domain within the protein, and how modification of different sites within the same protein can lead to diverse outcomes. Acetylation dynamics data, in combination with metabolite and inhibitor data, can further our understanding of the functional roles of specific acetyl sites and potentially aid in the development of therapeutics in aberrant acetylation associated diseases.

## Methods

### Sample Preparation

Four HeLa cell cultures were grown in suspension flasks until each reached a volume of 1 liter in Joklik Minimum Essential Medium (Sigma Aldrich, St. Loui, MO, USA) that had been supplemented with newborn calf serum (NCS)(Thermo Fisher Scientific, San Jose, CA, USA) to a final volume of 10%, Glutamax (Thermo Scientific) to a final concentration of 1X, and Penicillin/Streptomycin (Thermo Scientific) to a final concentration of 1X. For the 0 hour time point, 100 mL of each culture was collected at a cell confluence of about 4 × 10^5^ cells/mL. Then two cultures were transferred to ^13^C-glucose media and two cultures were transferred to D_3_-acetate media, each at a total volume of 1 L.

The ^13^C-glucose media was prepared following the Sigma Aldrich Joklik media for suspension cultures ingredients. However, instead of adding normal glucose, ^13^C-glucose was added at 2.5 g/L. Also, 2 mM of alanine was added to the media to reduce the incorporation of heavy labeled alanine into proteins^7^. The D_3_-acetate media was made in a similar manner, the only difference being that unlabeled glucose was added to the media as called for by the Sigma Aldrich Joklik component information list, and 10 mM D_3_-acetate was added as well. Each media preparation was filtered, and dialyzed fetal bovine serum (dFBS) was added to a final volume of 10%, Glutamax was added to a final concentration of 1X, and Penicillin/Streptomycin was also added to a final concentration of 1X.

After transferring two cultures to ^13^C-glucose media and two cultures to D_3_-acetate media, time points were collected at 0.5, 1, 4, 8, 12, 16, and 24 hours for each culture, keeping the overall amount of cells collected as close as possible to that of the 0 time point. Thus, there were two replicates for the cultures grown in ^13^C-glucose media and two replicates for the cultures grown in D_3_-acetate media. Each sample collected was washed twice with phosphate buffered saline (PBS) and the cell pellet was frozen at −80 °C until sample processing.

The protein extraction and digestion was conducted by lysing the cells in 750 μl of cold lysis buffer (6 M urea/2 M thiourea, 50 mM ammonium bicarbonate, pH 8.2, 1X Halt Protease Inhibitors Cocktail (Thermo Scientific), 10 mM sodium butyrate, 10 mM dithiothreital) by vortexing, followed by sonication. The samples were incubated 30 minutes at room temperature in lysis buffer following the sonication. Subsequently, samples were alkylated with 30 mM iodoacetamide (IAA) in the dark for 30 minutes at room temperature. Protein concentration was determined and 10 mg of protein from each sample was collected for further processing. Proteins were digested with endopeptidase Lys-C (Wako, MS grade) for 3 hours, after which the solution was diluted 6 times with 50 mM triethylammonium bicarbonate (TEAB). Proteins were then digested with trypsin (Promega, Madison, WI, USA) at an enzyme-to-substrate ratio of 1:50 for 12 hours at room temperature.

The samples were next desalted using Sep-Pak C18 Vac cartridges (Waters, Milford, MA, USA). The columns were washed with 3 mL of 100% acetonitrile (ACN), followed by a wash with 2 mL 75% ACN. The columns were equilibrated with 2 mL 0.1% trifluoroacetic acid (TFA). The sample was loaded and run through the column twice, eluted with 60% ACN + 0.1% TFA, and lyophilized.

The samples were enriched for acetylated peptides by immunoaffinity purification (IAP) using an anti-acetyl lysine antibody following the PTMScan protocol (Cell Signaling Technologies, Danvers, MA, USA). The lyophilized samples were resuspended in 1X IAP buffer (Cell Signaling Technologies). The antibody-bead slurry was washed with 1X PBS before transferring the peptide solution to the beads. The peptide solutions were incubated with the beads overnight at 4 °C while rotating. After incubation, the beads were washed with IAP buffer and with mass spectrometry grade water. The peptide samples were eluted from the beads using 0.15% TFA. The eluate was purified by stage-tipping, as described in other studies^50^, but with C8 filter paper. Samples were eluted from the stage-tips in 75% ACN + 0.1% TFA and lyophilized.

### Acetyl-CoA Metabolite Sample Preparation and Extraction

HeLa cells were grown in Joklik media and split into four cultures to create biological replicates for both the ^13^C-glucose labeling and the D_3_-acetate labeling. Time point samples were collected prior to transferring to ^13^C-glucose media or D_3_-acetate media, as well at 0.5, 1, 4, 8, 12, 16, and 24 hours after transferring the cultures to their respective heavy media.

Cell pellets from each time point were resuspended in a cold (−80 °C) solution of 80% methanol/20% H_2_O, and then incubated in a −80 °C freezer for 15 minutes. The suspension was centrifuged immediately at 3400 × g for 5 mins at 4 °C. After collecting the supernatant, the pellet was resuspended in 80% methanol/20% H_2_O, and the previous two steps were repeated. The two supernatants were dried in a speed vacuum, resuspended in water, and combined. Insoluble debris was pelleted by centrifugation prior to analysis by mass spectrometry.

### MS Analysis, Database Searching and Quantification

Dried samples were resuspended in buffer-A (0.1% formic acid) and loaded onto an Easy-nLC system (Thermo Scientific), coupled online with an Orbitrap Fusion Tribrid mass spectrometer (Thermo Scientific). Peptides were loaded into a picofrit 18 cm long fused silica capillary column (75 µm inner diameter) packed in-house with reversed-phase Repro-Sil Pur C18-AQ 3 µm resin. A gradient of 120 minutes total was set for peptide elution from 3-38% buffer B (80% ACN, 0.1% formic acid) for 100 minutes at a flow rate of 300 nl/min, followed by 38-98% buffer B for 15 minutes, and followed by 98% buffer B for 5 minutes. The MS method was set up in a data-dependent acquisition (DDA) mode. For full MS scan, the mass range of 350-1200 m/z was analyzed in the Orbitrap at 120,000 resolution (FWHM at 200 m/z) and 5 × 10e5 AGC target value. MS/MS was performed with the Quadrapole as the isolation mode using TopSpeed mode (3 seconds), with the Orbitrap as the detector. Dynamic exclusion was set to 60 seconds. HCD collision energy was set to 27, AGC target to 5e4 and maximum injection time to 200 msec.

MS raw files were analyzed by pFind^51^. MS/MS spectra were searched against the Human UniProt FASTA database [9606]. The modifications considered included acetylation and oxidation, and the fixed modification was carbamidomethylation. The enzyme was set to trypsin. For the Orbitrap, the precursor tolerance was set to 10 ppm, and the fragment tolerance was set to 0.02 Da. The data was searched against a decoy database, with the false discovery rate (FDR) set to 0.01.

The output file was filtered to remove any unmodified peptides, in order to obtain an identification list of acetylated peptides. This list of all acetylated peptides detected included the precursor m/z, charge state, retention time, search score, sequence, modification, and protein name for each acetylated peptide. Next for each acetylated peptide the isotopic cluster was obtained, including the monoisotopic peak (noted as M0), the M + 2 peak, and the M + 4 peak. The chromatographic profiles of these peaks were obtained, and the area under curve (AUC) was calculated. The influence of M0 on M + 2 was subtracted (on one-acetylation peptides, M + 2 peak is influenced). The influence of M + 2 on M + 4 was also subtracted (on two-acetylation peptides, M + 2 and M + 4 peaks are influenced). The sum of M + 2 AUC and M + 4 AUC divided by the sum of M0, M + 2, and M + 4 AUC was calculated as the heavy/(heavy + light) ratio.

Using an Agilent 1200 series liquid chromatography system (G1312 Bin, G1367 HiP-ALS), metabolite extracts were separated on a 100 × 2 mm Synergi 2.5 u HydroRP 100 A column (Phenomenex, Torrance, CA, USA) coupled to an LTQ Orbitrap mass spectrometer (Thermo Scientific). Chromatography conditions were 2% buffer B (100% methanol) in 98% buffer A (97% water, 3% methanol, 10 mM tributylamine, 15 mM acetic acid) for 1.5 minutes, increasing to 15% B over 1.5 mins, increasing to 95% B over 2.5 minutes, holding at 95% B for 9 minutes, decreasing to 2% B over 0.5 minutes, and holding at 2% B for the final 6 minutes. The flow rate was set to 0.2 ml/min. Full-scan mass spectra were acquired from 700-1000 m/z in negative profile mode with a resolution of 60,000. The top 3 most abundant ions were selected for CID fragmentation using an isolation width of 1.5 m/z, a normalized collision energy of 35, an activation time of 30 ms, and an activation Q of 0.250. MS raw files were analyzed using Skyline^52^.

Supplementary tables are provided and raw files are uploaded in Chorus (https://chorusproject.org/pages/index.html) under the project number 1351.

### Bioinformatics and Statistical Analysis

Reproducibility between the two biological replicates was assessed using Pearson correlation and correlation significance (t conversion). After converting the quantification values into ratios of heavy forms/total forms, the significance of monotonic increase of the ratio across the time points was assessed using the Mann-Kendall test on the average of the two biological replicates. Clustering analysis was performed using Euclidean trees with Perseus^42^. Gene Ontology enrichment analysis was performed using GOrilla^43^. Protein networks based on known protein-protein interactions was performed using the STRING^53^ database (v 10.0), and figure layout was modified with Cytoscape^54^ (v 3.3.0). Motif analysis was performed using pLogo^55^. Domain information to map acetylations within domains was retrieved from the UniProt database.

## Electronic supplementary material

Supplementary Figures

Supplementary Table S1

Supplementary Table S2
