# Supplementary material for: Proteome-wide acetylation dynamics in human cells
Source: Sci Rep. 2017 Aug 31;7:10296. doi: 10.1038/s41598-017-09918-3 (PMC5579049; doi:10.1038/s41598-017-09918-3)
Supplement: Supplementary file 1 — Supplementary Figures [file 41598_2017_9918_MOESM1_ESM.pdf]

Proteome-wide acetylation dynamics in human cells

Yekaterina Kori[1], Simone Sidoli[1], Zuo-Fei Yuan[1], Peder J. Lund[1], Xiaolu Zhao[2*] and Benjamin A. Garcia[1*]

[1]Epigenetics Institute, Department of Biochemistry and Biophysics, Perelman School of Medicine, University of Pennsylvania, Philadelphia, PA 19104, USA

[2]Hubei Key Laboratory of Cell Homeostasis, College of Life Sciences, Wuhan University, Wuhan, P.R.China, 430072

*Corresponding author:

Benjamin A. Garcia, Epigenetics Institute, Department of Biochemistry and Biophysics, Perelman School of Medicine, University of Pennsylvania, Room 9-124, 3400 Civic Center Blvd, Bldg 421, Philadelphia, PA 19104, USA. E-mail: bgarci@mail.med.upenn.edu; Phone: 1-215-573-9423; Fax: 215-573-4764

Xiaolu Zhao, Associate Professor, Room 6016, College of Life Sciences, Wuhan University, Luojiashan Road, Wuhan, P.R.China, 430072. Email: zhaoxiaolu@whu.edu.cn; Tel: + 86-27-68753797: Fax: + 86-27-68753797

A

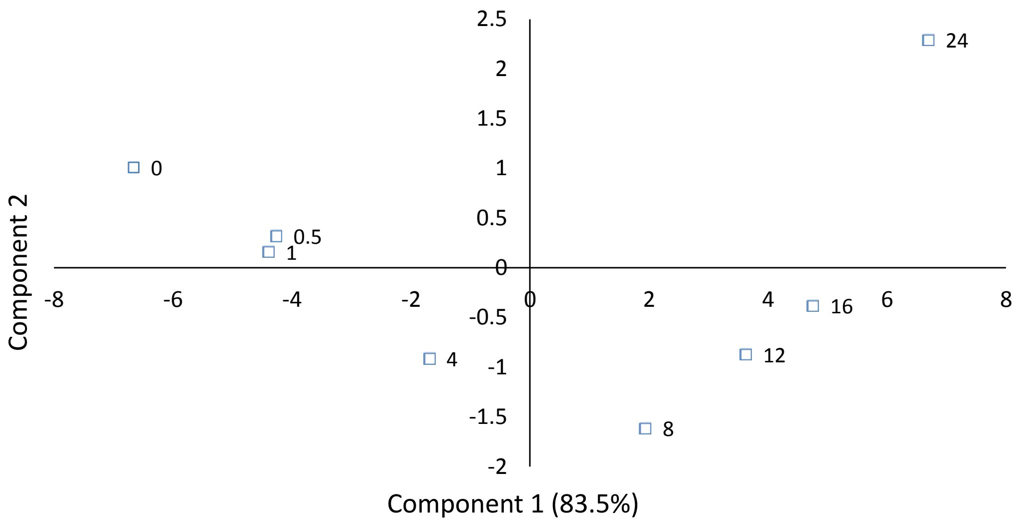

B

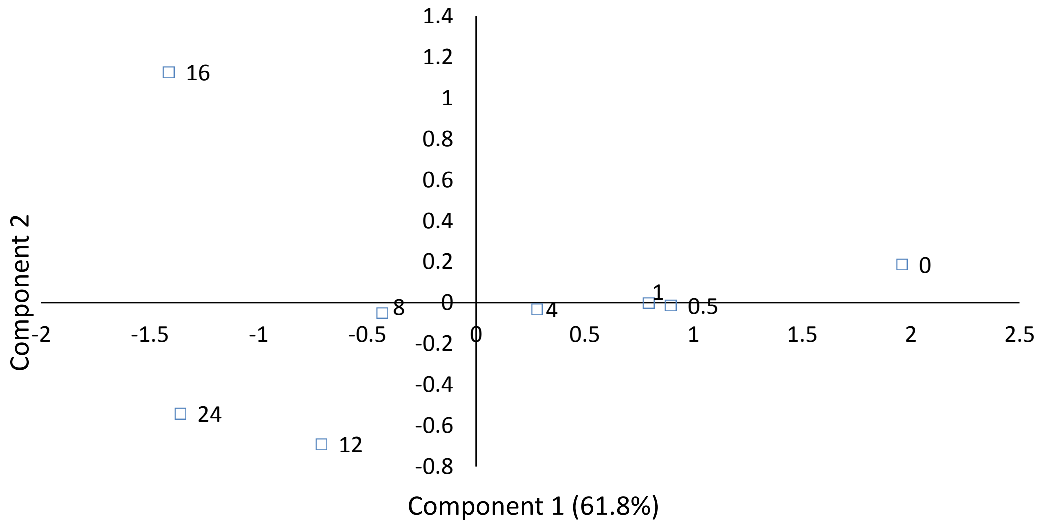

Supplementary Figure S1. Quality Control. A. The correlation between time points for the statistically significant peptides (average values) is plotted by principal component analysis (PCA) for the ^{13}C-glucose labeling experiment. B. The correlation between time points for the statistically significant peptides in the D_3-acetate labeling experiment (average values) are plotted by PCA.

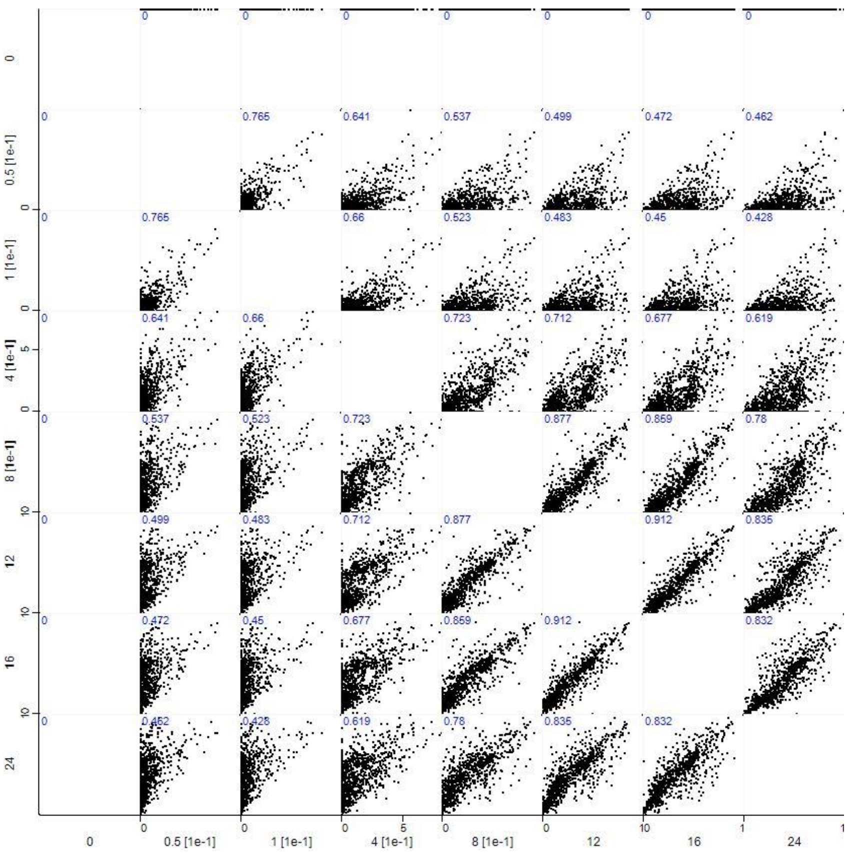

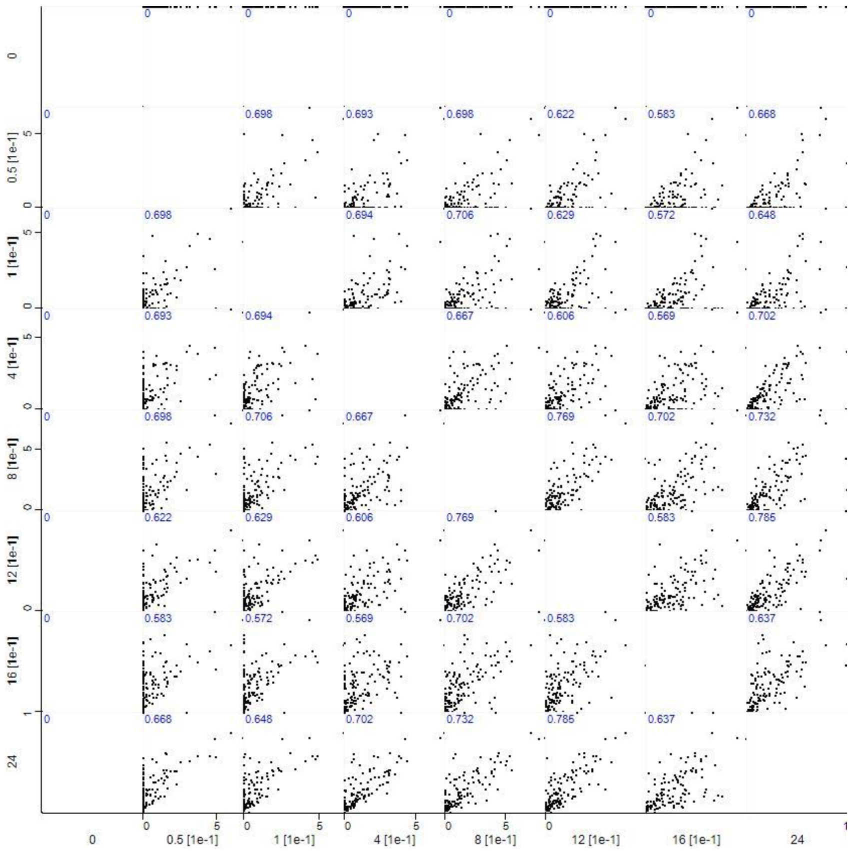

Supplementary Figure S2. Quality Control: Multi-Scatter Plots. A. The correlation between time points for the peptides with a statistically significant trend (average values) is plotted in a multi-scatter plot for the ^{13}C-glucose labeling experiment. B. The correlation between time points for the statistically significant peptides in the D_3-acetate labeling experiment (average values) are plotted by multi-scatter plot.

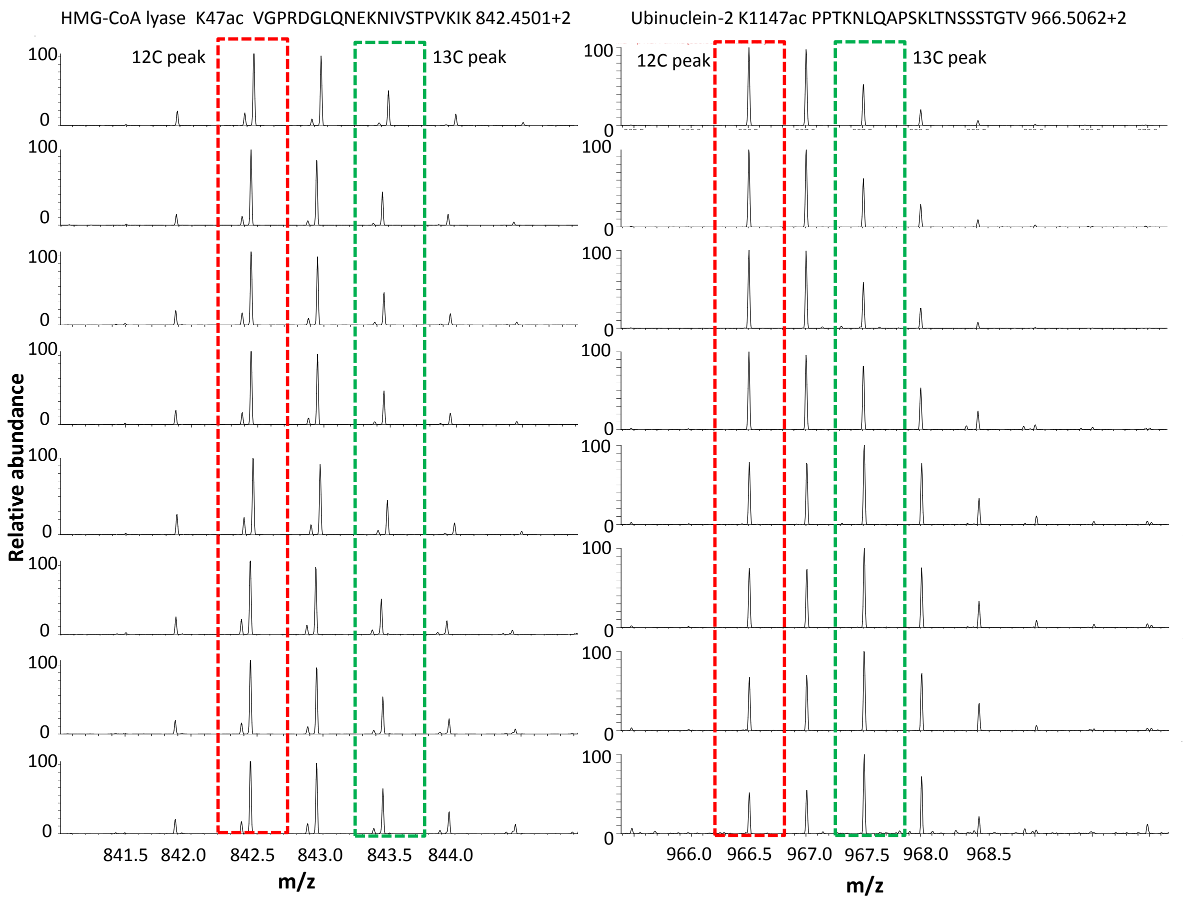

Supplementary Figure S3. Isotopic Patterns of Acetylation Incorporation Over Time. The mass spectra for two examples of acetylated peptides are shown at each time point, with the top spectra being 0 hours and the bottom-most spectra the 24 hours. The left peptide shows no incorporation of heavy acetylation, whereas the peptide on the right shows an increasing incorporation of heavy acetylation over time. The red dotted box indicates the monoisotopic peak, and the green dotted box indicates the heavy labeled peak.

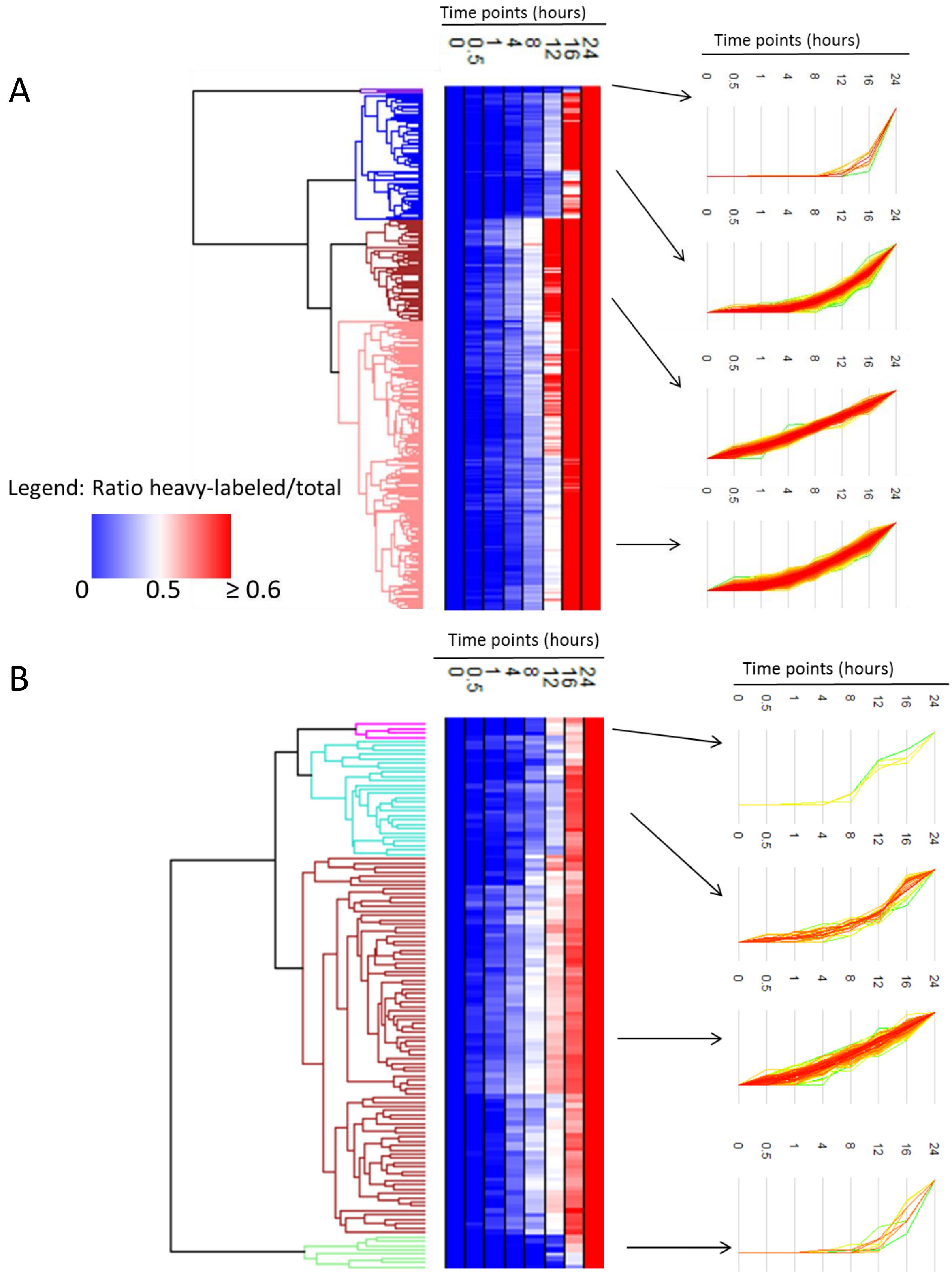

Supplementary Figure S4. Clustering of Heavy Acetylation Incorporation Trends. A. The trends obtained from the $^{13}C/(^{13}C+^{12}C)$ ratio for all the time points was transformed into a cumulative trend by normalizing each ratio (average between two replicates) to 100%, so that the 24 hour time point = 1. The heatmaps plot these cumulative values at each time point for the peptides with a statistically significant increasing trend detected in the ^{13}C-glucose labeled experiment. B. Heatmap of the cumulative values at each time point for the peptides with a statistically significant increasing trend detected in the D_3- acetate labeled experiment.

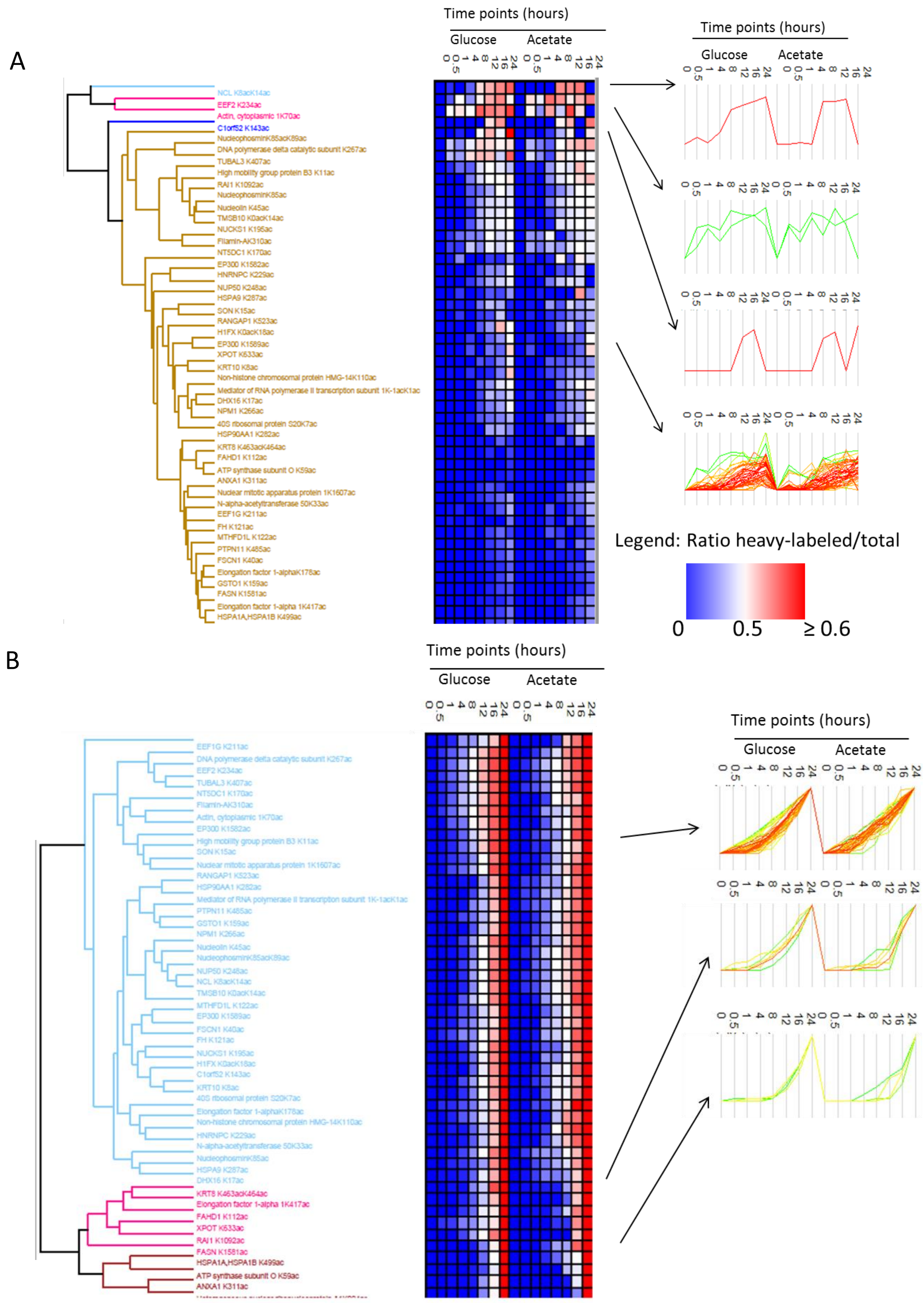

Supplementary Figure S5. Clustering of Trends for Sites Labeled by Both Glucose and Acetate. A. The heatmap demonstrates the $^{13}C/(^{13}C+^{12}C)$ ratio at each time point for the peptides that showed statistically significant heavy acetylation incorporation in both the ^{13}C-glucose labeled experiment and the D_3-acetate labeled experiment. B. The cumulative values of the peptides that showed statistically significant heavy acetylation incorporation in both the ^{13}C-glucose labeled experiment and the D_3-acetate labeled experiment are plotted.

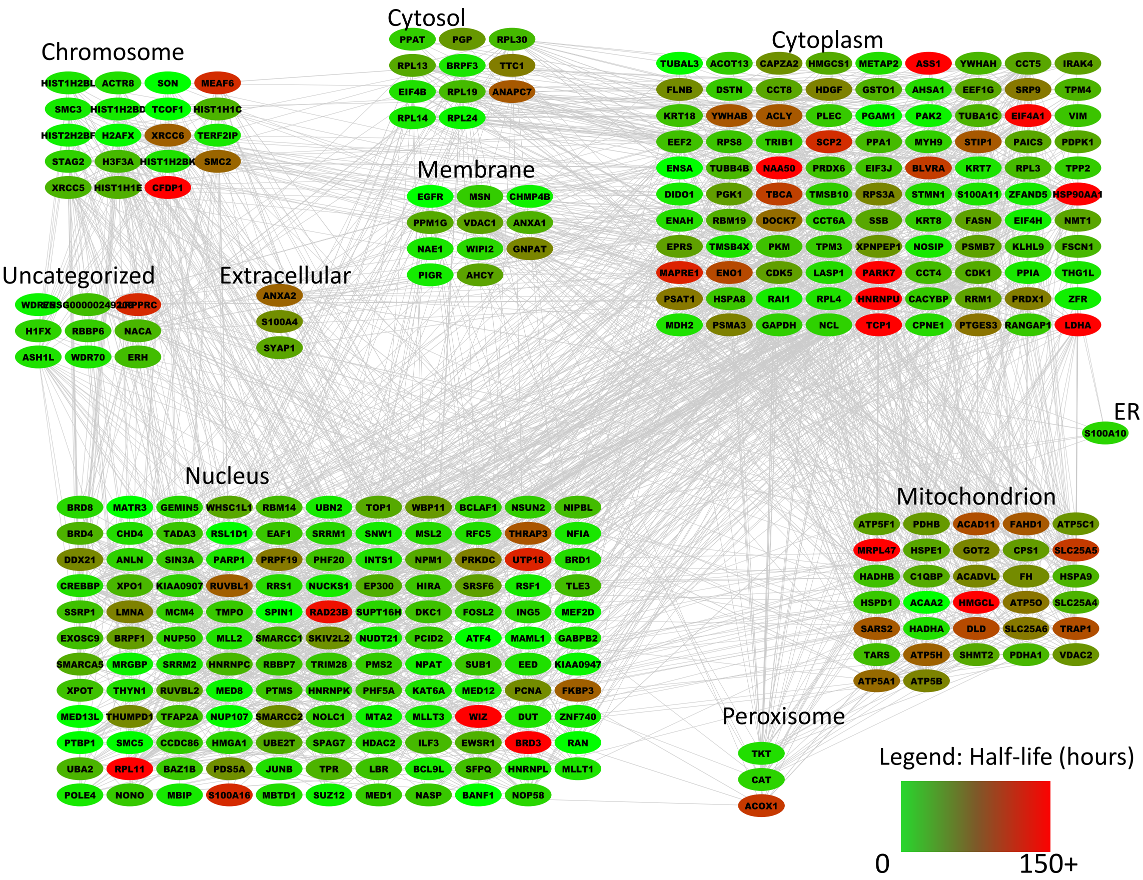

Supplementary Figure S6. Glucose-Labeled Networks. The map portrays the connections between proteins with acetyl sites identified in this study, by ^{13}C-glucose labeling. The average half-lives between all detected sites within a protein were calculated, and the average half-life is indicated by the color.

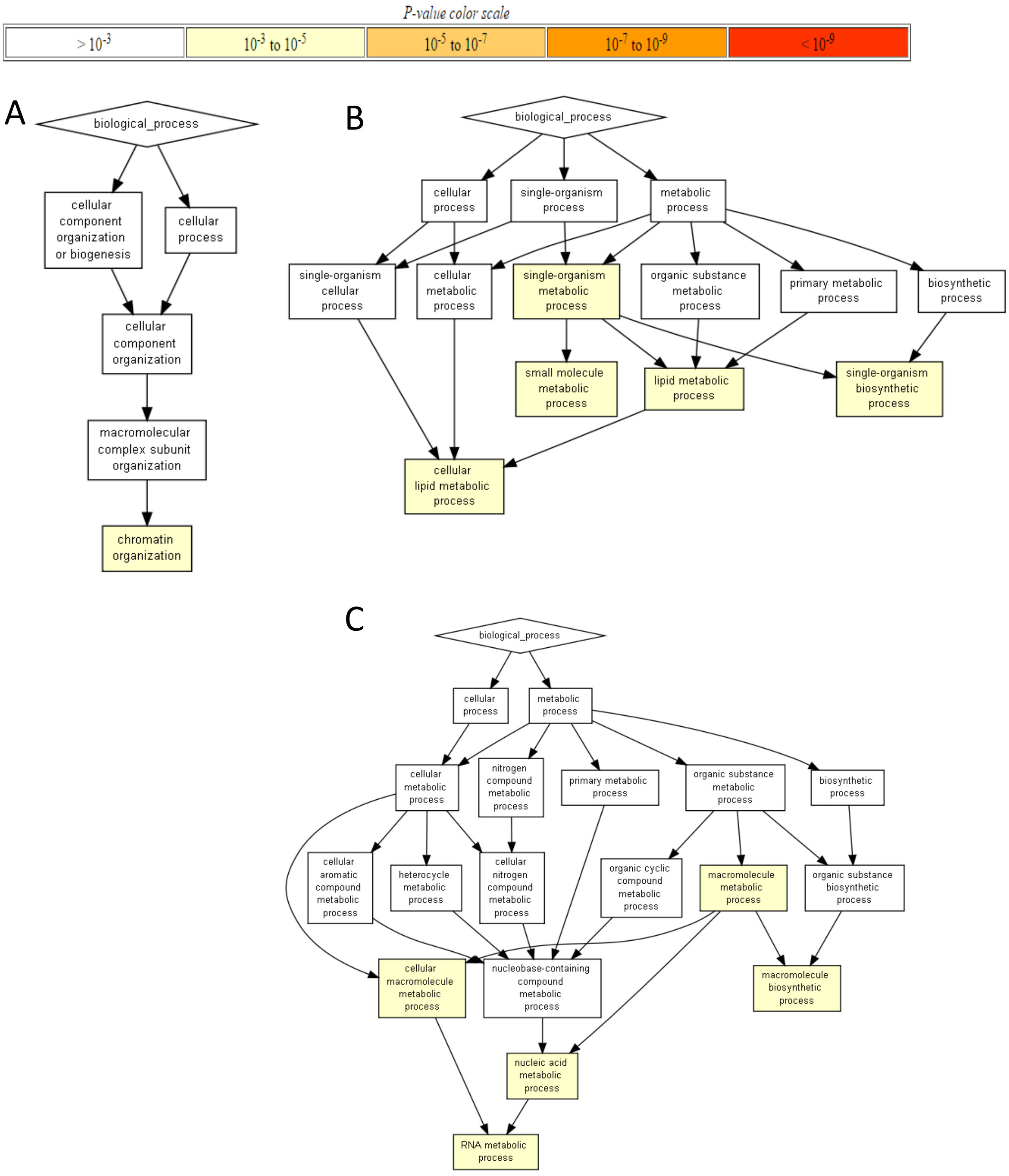

Supplementary Figure S7. Gene Ontology Enrichment. A. Gene ontology enrichment for the second fastest glucose heatmap cluster (Figure 4A, third from the top, turquoise blue cluster) in the ^{13}C-glucose labeled experiment. Only 30% of the terms were recognized for the analysis. B. Gene ontology enrichment for the slowest rate heatmap cluster in the ^{13}C-glucose labeled experiment (fourth from the top, light blue cluster). Only 30% of terms used in the analysis. C. Gene ontology enrichment for the medium rate heatmap cluster (bottom-most, dark blue cluster) in the ^{13}C-glucose labeled experiment. Only 20% of terms used in the analysis.

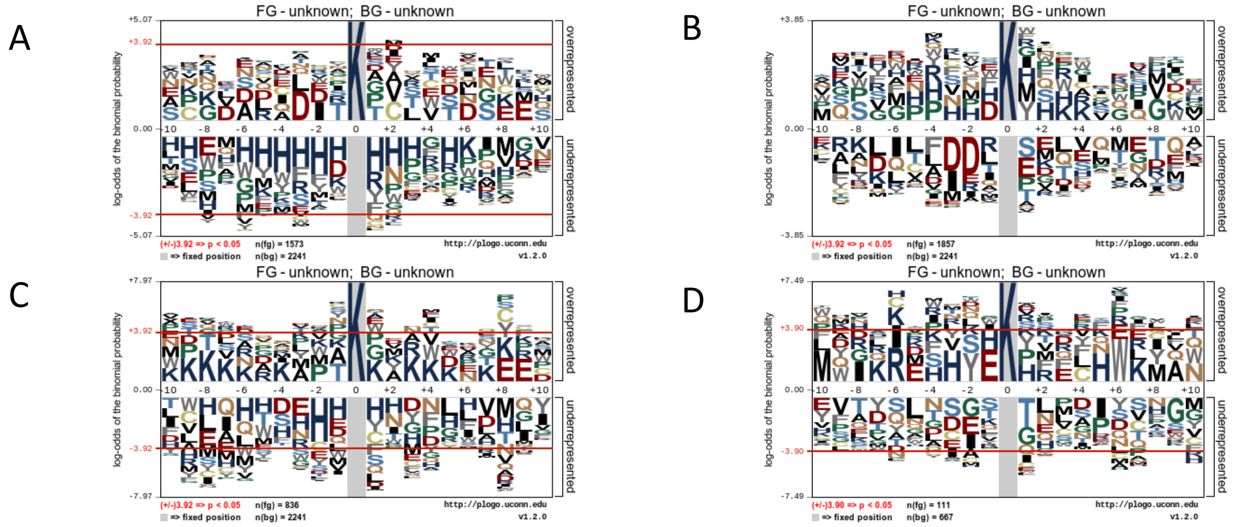

Supplementary Figure S8. General Motif Analysis. A. Motif analysis was conducted using pLogo. The sequences for all the acetylated peptides detected in the ^{13}C-glucose experiment were searched against a background of all the acetylated peptides detected by both glucose and acetate labeling. B. Acetylated peptides detected in the D_3-acetate labeled experiment against a background of all the acetylated peptides detected by both glucose and acetate labeling. C. Statistically significant (by Mann Kendall) acetylated peptides detected in the ^{13}C-glucose experiment against a background of all the acetylated peptides detected by ^{13}C-glucose labeling. D. Statistically significant acetylated peptides detected in the D_3-acetate experiment against a background of all the acetylated peptides detected by D_3-acetate labeling.

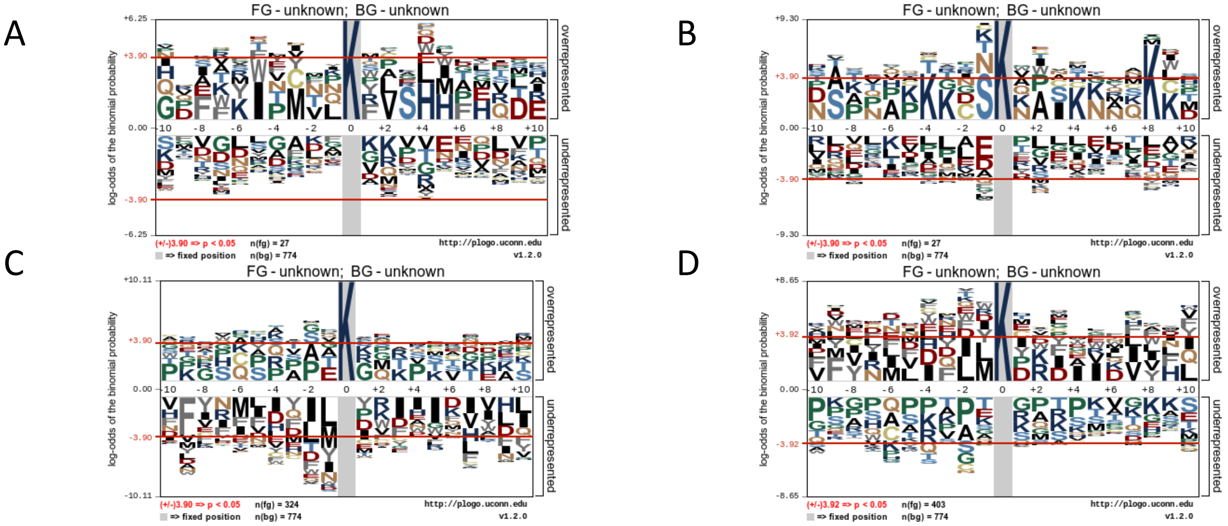

Supplementary Figure S9. Glucose Labeling Clusters Motif Analysis. A. The sequences for all the acetylated peptides in the fastest heatmap clusters (Figure 4A, the top two, red and pink clusters) in the glucose labeling experiment were searched against a background of the acetylated peptides detected by ^{13}C-glucose labeling that showed a statistically significant trend. B. The sequences for all the acetylated peptides in the third fastest heatmap clusters (the third from the top, turquoise blue cluster) in the glucose labeling experiment were searched against a background of the acetylated peptides detected by ^{13}C-glucose labeling that showed a statistically significant trend. There is a slight enrichment of K at the -4 position, with a frequency of 40.74%, S at the -1 position, with a frequency of 37.04%, and K at the +8 position, with a frequency of 55.56%. C. The sequences for all the acetylated peptides in the medium rate heatmap clusters (the bottom-most, dark blue cluster) in the glucose labeling experiment against a background of the acetylated peptides detected by ^{13}C-glucose labeling that showed a statistically significant trend. D. The sequences for all the acetylated peptides in the slowest rate heatmap clusters (the fourth from the top, light blue cluster) in the glucose labeling experiment against a background of the acetylated peptides detected by ^{13}C-glucose labeling that showed a statistically significant trend.

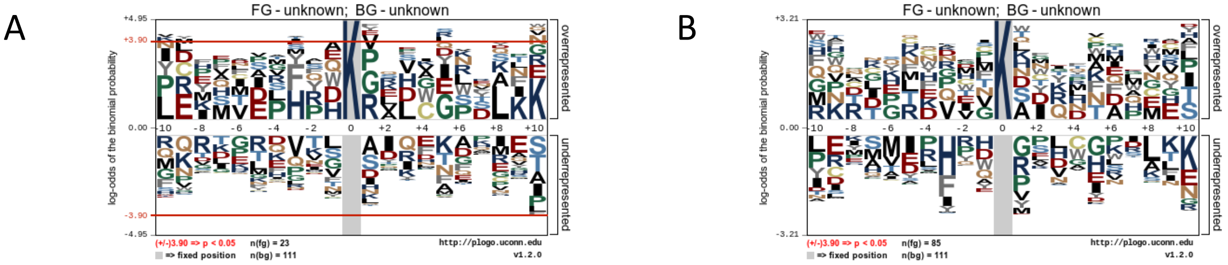

Supplementary Figure S10. Acetate Labeling Clusters Motif Analysis. A. The sequences for all the acetylated peptides in the medium rate heatmap clusters (Figure 4B, the middle, dark turquoise cluster) in the acetate labeling experiment against a background of the acetylated peptides detected by D_3-acetate labeling that showed a statistically significant trend. B. The acetylated peptides in the slowest rate heatmap clusters (the bottom-most, green and light blue clusters) in the acetate labeling experiment were searched against a background of the acetylated peptides detected by D_3-acetate labeling that showed a statistically significant trend.

Supplementary Table S1. Identified Acetyl Peptides and their Heavy Acetylation Ratios. The excel spreadsheet contains the identified acetyl sites, as well as the accession numbers, ENSP identifier, gene name, and description of the proteins they are found on. This data had been obtained after several quality control checkpoints, such as searching the data against a decoy database with the false discovery rate (FDR) set at 0.01. Additionally, a manual check was conducted of the MS/MS spectra for many of the acetylated peptides. The data for the glucose labeling is shown first, with the $^{13}C/(^{13}C+^{12}C)$ ratio at each time point is listed. The red header indicates data for replicate 1 and the green header indicates data for replicate 2. The final half-time is the calculated time to reach 50% heavy labeling. The correlation between the replicates is shown, with the p-value for the correlation in the next column over. The blue header indicates the average ratios between replicates at each time point. The MK test is the Mann Kendall statistical test for an increasing incorporation trend (p-value < 0.05 is significant). This is followed by data for acetate labeling, which is organized in the same manner.

Supplementary Table S2. Acetyl Peptides that Map to Specific Domains. The excel spreadsheet contains the identified acetyl sites, as well as the ENSP identifier, gene name, and description of the proteins they are found on. The column with the purple header indicates whether a specific acetyl site was mapped to a domain, zinc-finger region, general region, coiled coil, compositional bias, motif, or repeat. The following column contains a brief description of the domain/region, and the last column contains the residue span of the domain/region.
